# Supplementary figures and images for: Altered neural recruitment during single and dual tasks in athletes with repeat concussion
Source: Front Hum Neurosci. 2024 Dec 11;18:1515514. doi: 10.3389/fnhum.2024.1515514 (PMC11668694; doi:10.3389/fnhum.2024.1515514)

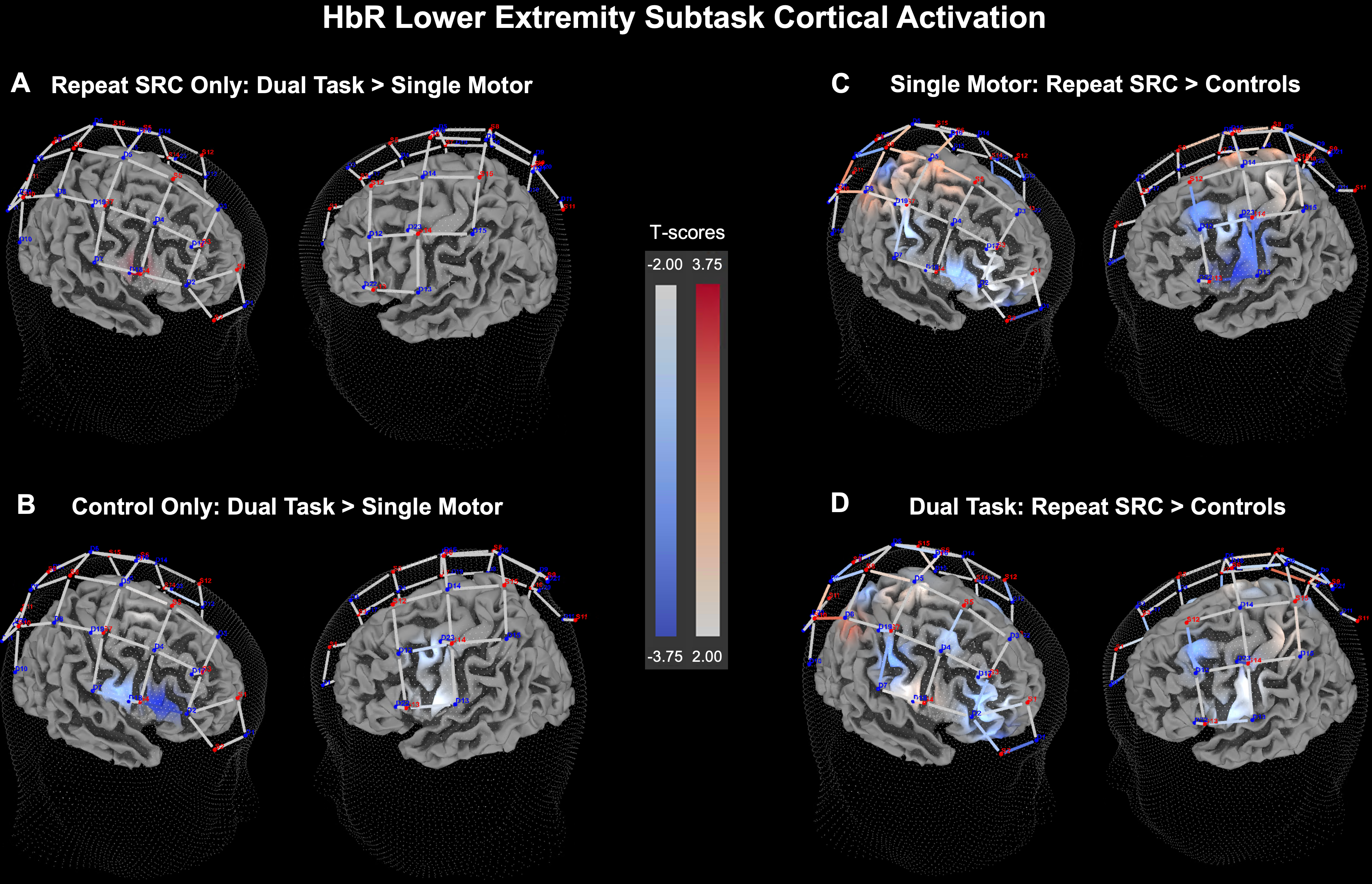

Supplement: Supplementary Figure S1 — Lower extremity (LE) neural recruitment significance map for HbR beta change. (A) Dual task activation contrasted against single motor task activation for the repeat SRC group only. (B) Dual task activation contrasted against single motor task activation for the control group only. (C) Activation in the repeat SRC group contrasted against activation of the control group for the single motor task condition. (D) Activation in the repeat SRC group contrasted against activation of the control group for the dual task condition. The colored bars illustrate a random-effects t-score map with red indicating increased activation and blue indicating decreased activation. [file Image_1.tiff]

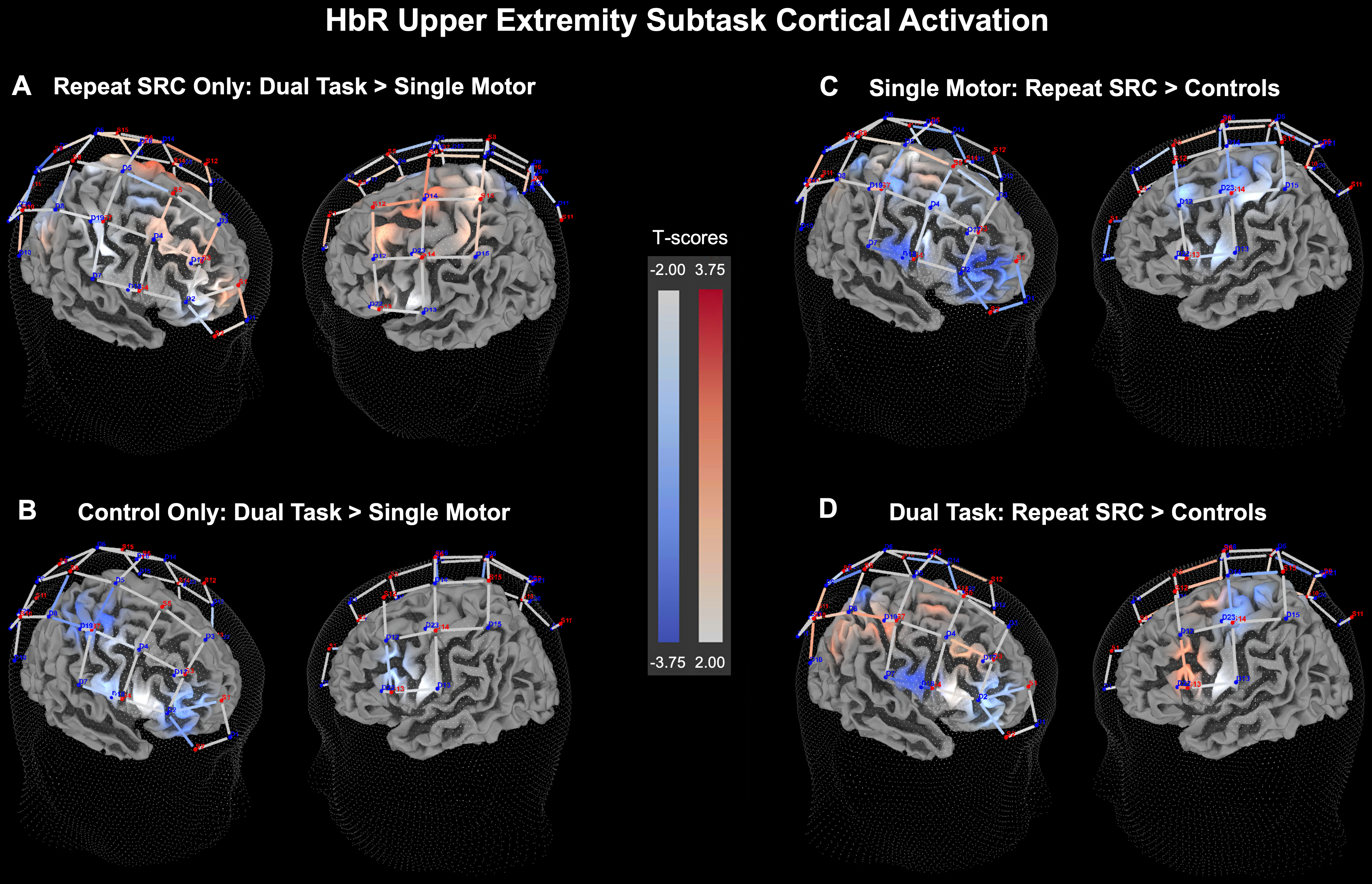

Supplement: Supplementary Figure S2 — Upper extremity (UE) neural recruitment significance map for HbR beta change. (A) Dual task activation contrasted against single motor task activation for the repeat SRC group only. (B) Dual task activation contrasted against single motor task activation for the control group only. (C) Activation in the repeat SRC group contrasted against activation of the control group for the single motor task condition. (D) Activation in the repeat SRC group contrasted against activation of the control group for the dual task condition. The colored bars illustrate a random-effects t-score map with red indicating increased activation and blue indicating decreased activation. [file Image_2.tiff]
